# The pathogenic Th profile of human activated memory Th cells in early rheumatoid arthritis can be modulated by VIP

**DOI:** 10.1007/s00109-014-1232-4

**Published:** 2014-11-28

**Authors:** Rebeca Jimeno, Rosa P. Gomariz, Marina Garín, Irene Gutiérrez-Cañas, Isidoro González-Álvaro, Mar Carrión, María Galindo, Javier Leceta, Yasmina Juarranz

**Affiliations:** 1 Departamento de Biología Celular, Facultad de Biología, Universidad Complutense de Madrid, 28040 Madrid, Spain; 2 División de Terapias Innovadoras en el S. Hematopoyético, CIEMAT/CIBERER, Unidad Mixta de Terapias Avanzadas CIEMAT/IIS Fundación Jiménez Díaz, Madrid, Spain; 3 Servicio de Reumatología, Instituto de Investigación Sanitaria Hospital La Princesa, Madrid, Spain; 4 Servicio de Reumatología, Instituto de Investigación Hospital 12 de Octubre (I+12), Madrid, Spain

**Keywords:** Rheumatoid arthritis, Th17, Th1, Vasoactive intestinal peptide, VPAC receptors

## Abstract

**Abstract:**

Our aim is to study the behavior of memory Th cells (Th17, Th17/1, and Th1 profiles) from early rheumatoid arthritis (eRA) patients after their in vitro activation/expansion to provide information about its contribution to RA chronicity. Moreover, we analyzed the potential involvement of vasoactive intestinal peptide (VIP) as an endogenous healing mediator. CD4^+^CD45RO^+^ T cells from PBMCs of HD and eRA were activated/expanded in vitro in the presence/absence of VIP. FACS, ELISA, RT-PCR, and immunocytochemistry analyses were performed. An increase in CCR6^+^/RORC^+^ cells and in RORC-proliferating cells and a decrease in T-bet-proliferating cells and T-bet^+^/RORC^+^ cells were shown in eRA. mRNA expression of IL-17, IL-2, RORC, RORA, STAT3, and Tbx21 and protein secretion of IL-17, IFNγ, and GM-CSF were higher in eRA. VIP decreased the mRNA expression of IL-22, IL-2, STAT3, Tbx21, IL-12Rβ2, IL-23R, and IL-21R in HD and it decreased IL-21, IL-2, and STAT3 in eRA. VIP decreased IL-22 and GM-CSF secretion and increased IL-9 secretion in HD and it decreased IL-21 secretion in eRA. VPAC_2_/VPAC_1_ ratio expression was increased in eRA. All in all, memory Th cells from eRA patients show a greater proportion of Th17 cells with a pathogenic Th17 and Th17/1 profile compared to HD. VIP is able to modulate the pathogenic profile, mostly in HD. Our results are promising for therapy in the early stages of RA because they suggest that targeting molecules involved in the pathogenic Th17, Th17/1, and Th1 phenotypes and targeting VIP receptors could have a therapeutic effect modulating these subsets.

**Key messages:**

- Th17 cells are more important than Th1 in the contribution to pathogenesis in eRA patients.
- Pathogenic Th17 and Th17/1 profile are abundant in activated/expanded memory Th cells from eRA patients.
- VIP decreases the pathogenic Th17, Th1, and Th17/1 profiles, mainly in healthy donors.
- The expression of VIP receptors is reduced in eRA patients respect to healthy donors, whereas the ratio of VPAC_2_/VPAC_1_ expression is higher.

## Introduction

Immune memory facilitates the maintenance of health by preventing repeated infections but, if it becomes deregulated, it can also lead to chronic inflammation. Rheumatoid arthritis (RA) has been characterized by a Th1 response [1]. However, this description has been modified with the discovery of a new Th subset, Th17, which play a key role in inflammatory and autoimmune diseases, including RA [1]. Pro-inflammatory cytokine IL-17 is the signature cytokine of Th17 cells, but can also be secreted by non-immune cells [2]. Studies exploring the neutralization of IL-17 by antibodies or using IL-17-deficient mice showed that this cytokine is involved in the autoimmune collagen-induced arthritis (CIA) model [3]. In humans, existing studies suggest that Th17 cells and their related cytokines play an important role in the pathogenesis of RA and its number in peripheral blood is associated with disease activity [4]. Moreover, IL-17 levels are increased in the synovial fluid of RA patients [5]. This cytokine, through its specific receptors, is able to modulate the function of other cells in the joint such as fibroblast-like synoviocytes (FLS), macrophages, chondrocytes, and osteoclasts [3, 5, 6]. Thereby, IL-17 is a key orchestrator of RA chronicity. In addition to IL-17, other molecules have been tested as markers of the occurrence of Th17 cells in RA, such as the transcription factor RORC, the major lineage-specifying transcription factors for Th17 subset development [7], and the chemokine receptor CCR6, the characteristic Th17 homing receptor [8]. It has been described that in both peripheral blood of healthy donors (HD) and synovial fluid of RA patients, all IL-17-producing T cells expressing RORC were CCR6^+^ [9]. In addition, other transcription factors are strongly associated with the Th17 subset, such as RORA or STAT3 [1, 7].

Recent evidence suggests that Th17 cells may exhibit a pathogenic or non-pathogenic phenotype according to their cytokine secretion profile [10, 11]. Pathogenic Th17 cells secrete IL-17, IL-21, IL-22, IL-2, IFNγ, and GM-CSF and non-pathogenic Th17 cells secrete IL-17, IL-21, IL-9, and IL-10. This heterogeneity of Th17 cells is barely known in human RA. Moreover, epigenetic studies have shown that the Th17 subset is a less committed lineage when compared to Th1 and Th2 cells [1]. Th17 cells are reported to exhibit a high degree of phenotypic instability and plasticity, which enable them to acquire a Th1-like phenotype [12]. In RA patients, a strong association between Th17 and Th1 subsets has been shown, being also described the presence of a Th17/1 intermediate subset [13, 14], which belongs to the pathogenic Th17 phenotype.

As Th17 and Th1 are important in the pathogenesis of RA, the study of the effect of microenvironment mediators could be important to the design of therapies for their modulation. Vasoactive intestinal peptide (VIP), one of the best-studied immunomodulatory neuropeptides [15, 16], is secreted by lymphocytes and FLS in the joint [17]. VIP is involved in a broad range of functions through its binding to its specific receptors, VPAC_1_ and VPAC_2_ [18]. Healing effects of VIP in animal models of inflammatory/autoimmune diseases, including a decrease of Th1 and Th17 profiles, have been reported [15, 16, 19, 20]. In vitro studies have shown that VIP induces Th17 differentiation [21–23]. Specifically, it has been described that VIP prevents arthritis in a CIA model through its anti-inflammatory and immunomodulatory actions [19]. There are also evidences for VIP therapeutic effects in human RA [16, 17].

To date, studies on the involvement of Th17 and Th1 cells in RA pathology have analyzed their presence in blood or in synovial fluid of patients. However, little is known about the behavior of memory Th cells after their in vitro activation/expansion in early RA (eRA) that may provide information about the involvement of these cells in RA chronicity. Our aim is to examine, for the first time, in HD and in eRA patients the resulting phenotype after 7 days of in vitro activation/expansion of memory Th cells, analyzing both the Th17/1 and Th1 profiles and the specific profile and pathogenicity of Th17 cells. Moreover, we examine the role of VIP in the modulation of Th1, Th17/1, and Th17 phenotypes, studying the involvement of its receptors.

## Methods

### Patients

Samples from 13 HD to 14 eRA patients were included in this study. The study was performed according to the recommendations of the Declaration of Helsinki and was approved by the ethics committees of the Transfusion Center of *Comunidad Autónoma de Madrid* (CAM) and *La Princesa* and *12 de Octubre* hospitals (Madrid). Only data from patients fulfilling the 2010 ACR/EULAR criteria for eRA were collected [24]. Blood samples were collected previous to treatment prescription. HD were recruited from the Transfusion Center. Following the Spanish Personal Data Protection law, their demographic information was confidential. Among eRA patients, there were 2 males and 12 females, 71.4 % tested positive for ACPA, the mean age was 56.4 ± 4.2 years (mean ± SD), and the mean DAS28 was 4.1 ± 0.6 (mean ± SD).

### Isolation of human peripheral blood memory T cells

Memory Th cells were isolated from whole blood from HD and eRA patients. For mononuclear cell isolation, density gradient centrifugation by Ficoll–Hypaque (Sigma Aldrich) was used. CD4^+^ T cells were isolated by negative selection using a CD4^+^ T Cell Isolation Kit II (Miltenyi Biotec). CD4^+^CD45RO^+^ T cells were then isolated by negative selection using CD45RA^+^ MicroBeads (Miltenyi Biotec). The purity of CD4^+^CD45RO^+^ T cells was greater than 92 %.

### In vitro expansion of human memory T cells

CD4^+^CD45RO^+^ T cells were cultured at 10 × 10^4^ and at 5 × 10^4^ cells/well (for HD and eRA patients, respectively) in RPMI-1640-GlutaMAX media (Life Technologies, Carlsbad, CA, USA) supplemented with 10 % fetal bovine serum (Lonza, Basel, Switzerland) and 1 % penicillin/streptomycin (Life Technologies). Cells were activated/expanded with anti-CD3/anti-CD28 coated beads (Life Technologies). CD4^+^CD45RO^+^ T cells were cultured in the absence or presence of 10nM of VIP (Polypeptide group, Strasbourg, France) for both HD and eRA patients.

### RNA extraction and semi-quantitative real-time PCR

For total RNA extraction we used the TriReagent method (Sigma Aldrich, St. Louis, MO, USA). Two micrograms RNA were reverse transcribed using a High Capacity cDNA Reverse Transcription Kit (Life Technologies). Semi-quantitative RT-PCR analysis for all molecules tested was performed using TaqMan Gene Expression Master Mix (Life Technologies), with the exception of IL-22, which was tested using SYBR® Green PCR Master Mix (Life Technologies) [24]. β-actin was used as an endogenous reference gene. We normalized each sample with β-actin, using the formula 2^−∆∆Ct^. Amplification was performed in a 7900HT Fast Real-Time PCR System apparatus (Applied Biosystems, Waltham, MA, USA).

### Determination of secreted cytokines by ELISA

Cells were restimulated on day 7 with 20 ng/ml phorbol myristate acetate (PMA) and 0.5 μM ionomycin (Sigma Aldrich) for 6 h. The levels of IL-17A, IL-21, IL-22, IFNγ, IL-9 (eBioscience, San Diego, CA, USA), and IL-10 (Diaclone, Madrid, Spain) in supernatants were analyzed by ELISA. Final values were corrected considering the final volume of the cultures and the number of viable cells in each sample.

### Flow cytometry analysis

After 7 days of culture, cells were collected and labeled with phycoerythrin-conjugated CCR6 (clone 11A9, BD Pharmingen, San Jose, CA, USA). Cells were then fixed and permeabilized with Transcription Factor Buffer Set (BD Pharmingen) according to the manufacturer’s specifications. Next, cells were labeled with Alexa Fluor 488 conjugated T-bet (Clone O4 46, BD Pharmingen), Allophycocyanin-conjugated RORC (clone AFKJS-9, eBioscience), and Brilliant Violet 711-conjugated Ki67 (clone Ki-67, BioLegend, San Diego, CA, USA). Auto-fluorescence and isotype controls were set up to define non-specific fluorescence. Cytometric analysis was performed using a LSR Fortesa flow cytometer (Becton Dickinson, Franklin Lakes, NJ, USA, using BD FACSDiva software). Data analysis was performed using FCS Express v3 (De Novo Software).

### Immunocytochemistry staining

On day 7, cell suspensions were centrifuged onto glass slides, dried, and fixed. After rehydration and blocking, cells were incubated with 0.02 mg/mL rabbit anti-VPAC_1_ polyclonal antibody and mouse anti-VPAC_2_ monoclonal antibody (Acris Antibodies, San Diego, CA, USA). Cells were then incubated with 1 μg/mL Alexa Fluor 488 donkey anti-rabbit IgG and Alexa Fluor 594 goat anti-mouse IgG antibodies (Life Technologies) and counterstained with 1 μg/ml Hoechst. Fluorescence was examined on an Olympus BX51 microscope with DP72 camera model (Olympus) and a Leica SP-2 AOBS confocal microscope with inverted stand Leica DM IRE2 (Leica).

### Statistical analysis

Samples were tested with a Normality test. A *t*-test was used to compare different groups and correlations were conducted using Pearson’s coefficient test. Both statistical tests were done using GraphPad Prism version 4.0 software (GradphPad Software).

## Results

### Th17 and Th1 phenotypes in eRA patients versus HD

**Fig. 1:**
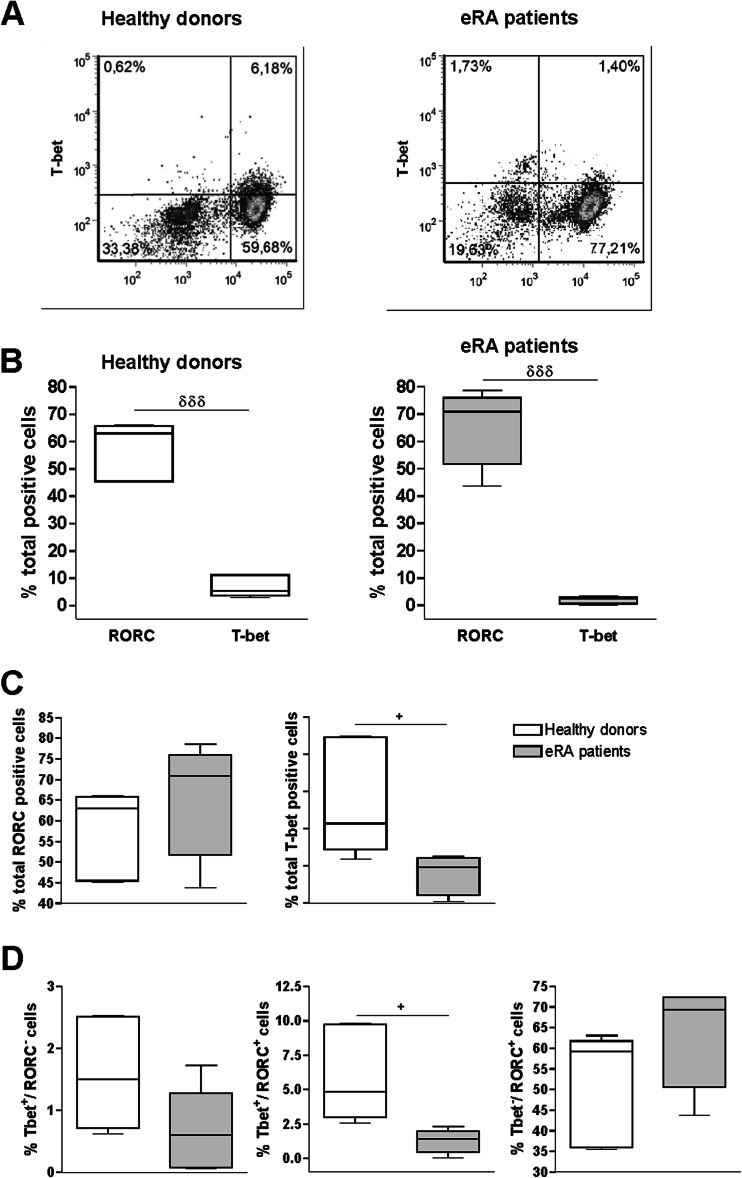
Th17 and Th1 phenotypes of activated/expanded memory Th cells from eRA patients and healthy donors. T-bet and RORC expression was determined by flow cytometry analysis in memory Th cells after activation/expansion for 7 days. Auto-fluorescence and isotype controls were set up to determine the non-specific fluorescence signal. **a** Figure shows a representative dot plot analysis indicating T-bet/RORC expression. **b**–**c** Figure shows the percentage of total RORC positive cells, total T-bet-positive cells. **d** Figure shows the percentage of T-bet^+^/RORC^−^ cells, T-bet^+^/RORC^+^, and T-bet^−^/RORC^+^ cells. Data are the mean ± SEM of three different cultures, performed in triplicate. Differences between RORC and T-bet expression in CD4^+^CD45RO^+^ T cells were statistically significant, ^δδδ^ *P* < 0.001. Differences between CD4+CD45RO+T cells from HD and eRA patients were statistically significant, ^+^ *P* < 0.05

We analyzed the presence of Th17 (T-bet^−^/RORC^+^), Th1 (T-bet^+^/RORC^−^), and Th17/1 (T-bet^+^/RORC^+^) cells by flow cytometry after 7 days of in vitro activation/expansion of Th cells from eRA patients and HD (Fig. 1). Results showed that RORC^+^ cells were more abundant than T-bet^+^ cells in both HD and eRA patients (Fig. 1b). Percentage of total RORC^+^ cells was higher, although not significantly, in eRA patients than in HD, whereas the percentage of total T-bet^+^ cells was lower (Fig. 1c). Percentage of T-bet^+^/RORC^+^ cells was significantly lower in eRA patients (Fig. 1d).

### Pathogenic Th17 phenotype in eRA patients versus HD

**Fig. 2:**
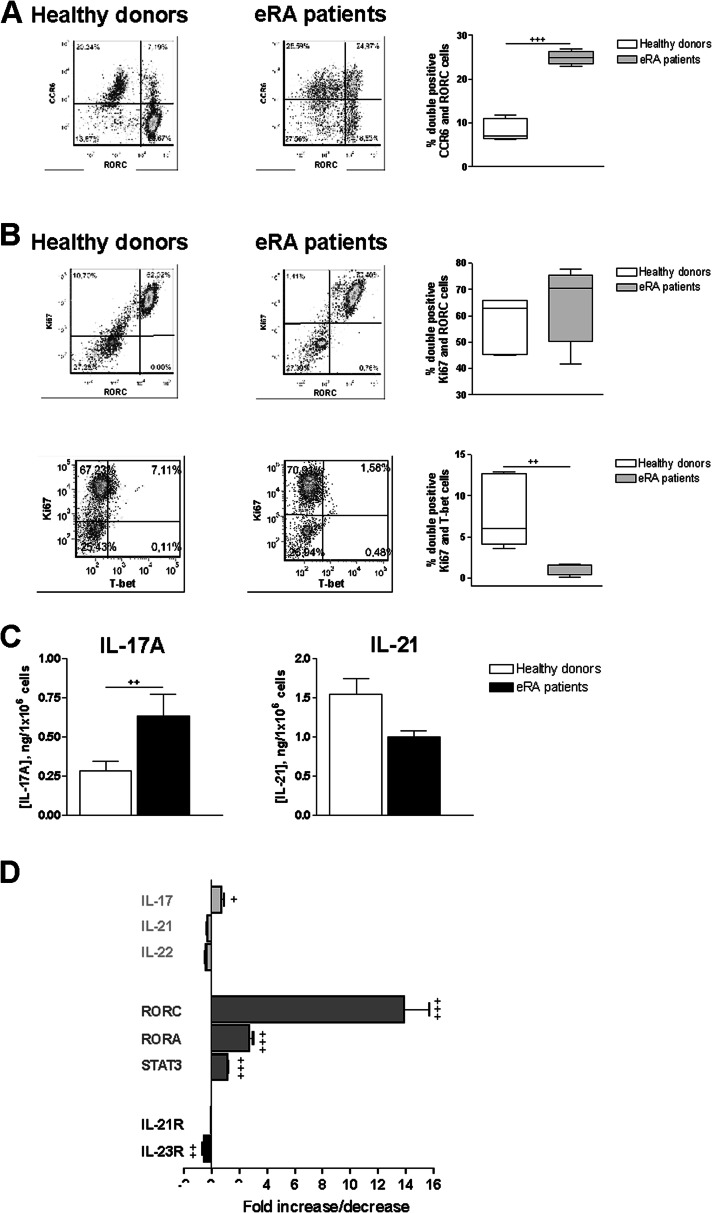
Th17 profile of activated/expanded memory Th cells from eRA patients and healthy donors. **a** CCR6 and RORC expression was determined by flow cytometry analysis in memory Th cells after activation/expansion for 7 days. Auto-fluorescence and isotype controls were set up to determine the non-specific fluorescence signal. The figure shows a representative dot plot analysis indicating CCR6/RORC expression. The percentage of CCR6/RORC double-positive cells was quantified. Data are the mean ± SEM of three different cultures, performed in triplicate. **b** Ki67/RORC and Ki67/T-bet expression were determined by flow cytometry analysis in memory Th cells after activation/expansion for 7 days. Auto-fluorescence and isotype controls were set up to determine the non-specific fluorescence signal. The figure shows two representative dot plot analysis indicating Ki67/RORC and Ki67/T-bet expression. Percentage of Ki67/RORC and Ki67/T-bet double-positive cells was quantified. Data are the mean ± SEM of three different cultures, performed in triplicate. **c** Protein expression of IL-17A and IL-21 was analyzed in culture supernatants by ELISA on day 7 of culture. Data are the mean ± SEM of eight different cultures, performed in duplicate. **d** mRNA expression of cytokines (*light grey*), transcription factors (*dark grey*), and cytokine receptors (*black*) was determined by real-time PCR in activated/expanded memory Th cells on day 7 of culture, after PMA and ionomycin stimulation. Data were analyzed normalizing with β-actin mRNA expression and compared with the mRNA expression of CD4^+^CD45RO^+^ T cells from HD. The fold change of each mRNA expression with respect to different conditions is represented. Data are the mean ± SEM of eight different cultures performed in triplicate. Differences between CD4^+^CD45RO^+^ T cells from HD and eRA patients were statistically significant, ^+^ *P* < 0.05, ^++^ *P* < 0.01, ^+++^ *P* < 0.001

Given the high frequency of Th17 cells in both HD and eRA patients we studied deeply their Th17 population. First, we characterized the Th17 subset through the expression of RORC and CCR6. Flow cytometric analysis of CCR6 and RORC double-expressing cells showed a significant increase in eRA (Fig. 2a). Then, we determined the cell proliferation by analysis of Ki67 expression by flow cytometry in RORC-expressing cells. As shown in Fig. 2b, eRA patients have a slight increase of Ki67^+^RORC^+^ cells compared to HD. However, the percentage of Ki67^+^/T-bet^+^ cells were significantly decreased in eRA patients. Next, we checked the secretion of IL-17 and IL-21 in culture supernatants. Memory Th cells from eRA patients showed significantly higher levels of IL-17 (Fig. 2c). In addition, we characterized by RT-PCR the expression of cytokines, transcription factors, and cytokine receptors of the Th17 profile. IL-17A, RORC, RORA, and STAT3 expression were higher in eRA patients, whereas IL-23R was lower than in HD (Fig. 2d). Taken together, these results indicate that activated/expanded memory Th cells from eRA patients are more committed towards the Th17 subset.

**Fig. 3:**
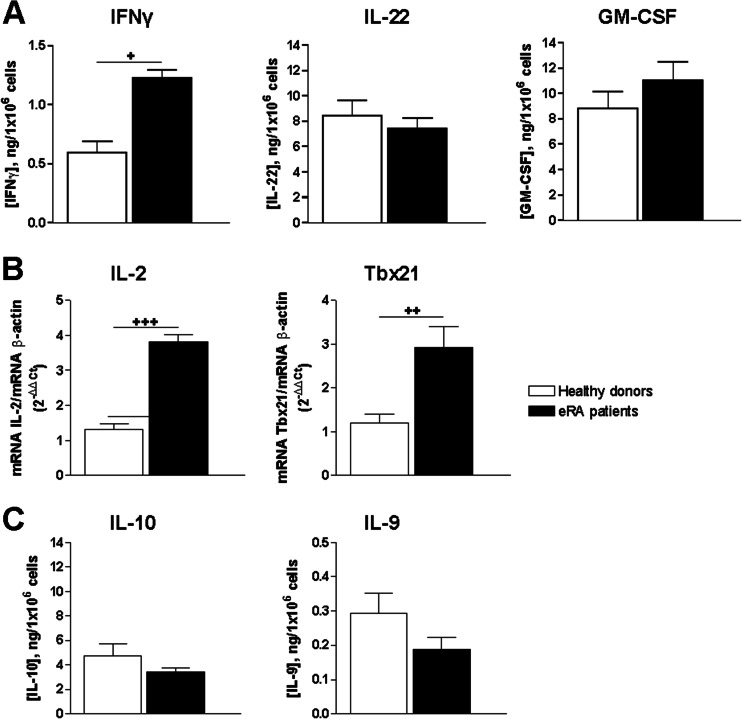
Pathogenic Th17 and Th1 phenotypes of activated/expanded memory Th cells from eRA patients and healthy donors. **a** Protein expression of IFNу, IL-22, and GM-CSF was analyzed in culture supernatants by ELISA after 7 days of expansion/activation of memory Th cells and after PMA and ionomycin stimulation. Data are the mean ± SEM of eight different cultures, performed in duplicate. **b** IL-2 and Tbx21 mRNA expression was determined by RT-PCR on day 7 of culture after PMA and ionomycin stimulation. Data were analyzed normalizing with β-actin mRNA expression and compared with the mRNA expression of CD4^+^CD45RO^+^ T cells from healthy donors. **c** Protein expression of IL-10 and IL-9 was analyzed in culture supernatants by ELISA after 7 days of expansion/activation of memory Th cells and after PMA and ionomycin stimulation. Data are the mean ± SEM of eight different cultures, performed in duplicate. Differences between CD4^+^CD45RO^+^ T cells from HD and eRA patients were statistically significant, ^+^ *P* < 0.05, ^++^ *P* < 0.01, ^+++^ *P* < 0.001

Next, we studied the pathogenic or non-pathogenic Th17 phenotype and the Th17/1 and Th1 profiles in eRA patients and HD. Cells from eRA patients secreted higher levels of IFNγ than HD (Fig. 3a). In addition, mRNA expression of IL-2 and Tbx21 (gene encoding T-bet protein, the lineage-specifying transcription factor for Th1) was higher in eRA patients (Fig. 3b). On the other hand, the levels of cytokines associated with a non-pathogenic phenotype IL-10 and IL-9 were slightly decreased in eRA patients, although not significantly (Fig. 3c). Together, these data indicate that activated/expanded memory Th cells from eRA patients show a more pathogenic Th17 and Th1 profile than memory Th cells from HD.

### VIP modulation of pathogenic Th phenotype in eRA patients versus HD

**Fig. 4:**
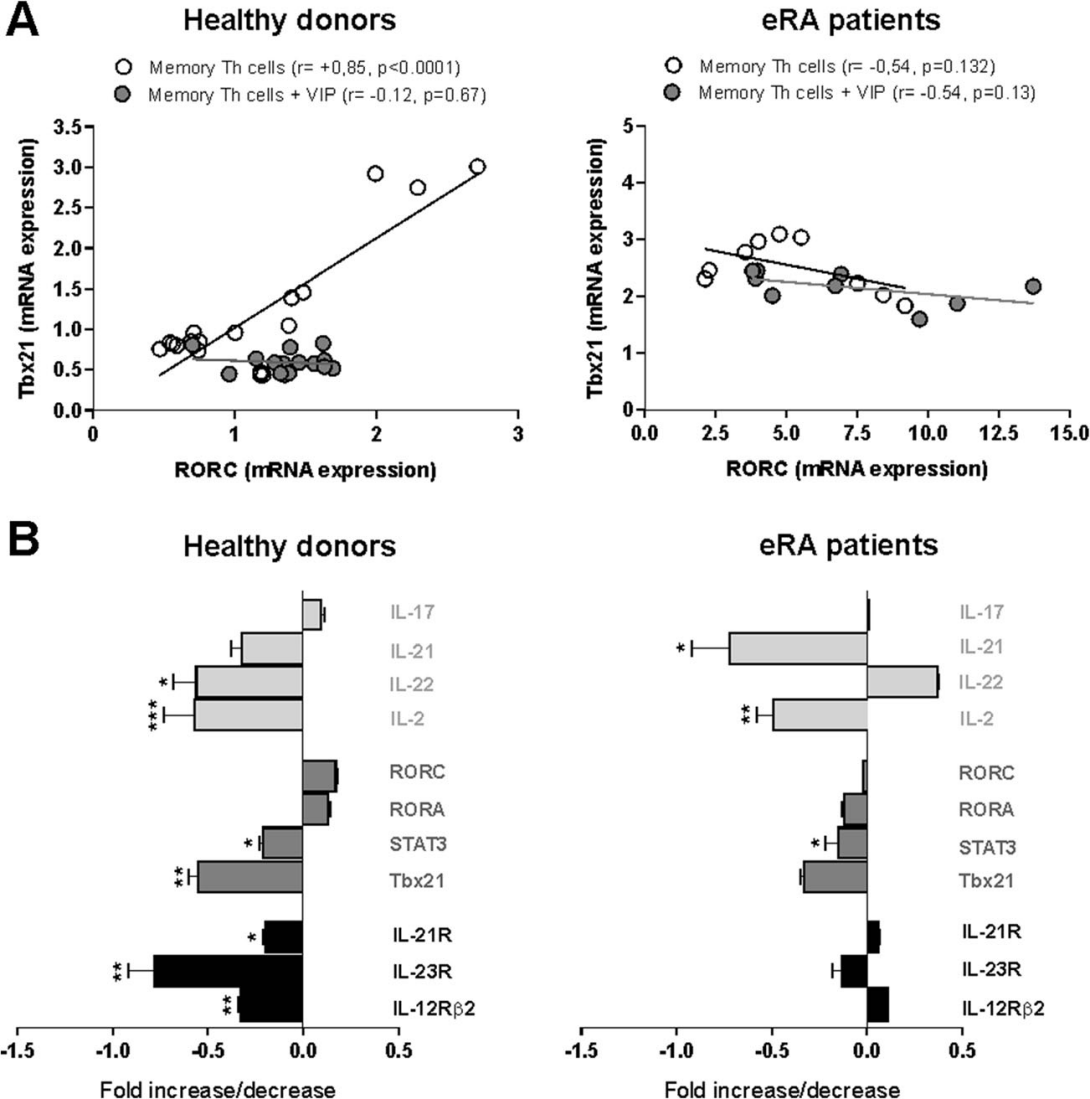
VIP modulation of pathogenic Th17 and Th1 phenotypes of memory T cells from eRA patients and healthy donors. **a** Correlation between mRNA expression of RORC and Tbx21 was determined using Pearson’s coefficients tests. Data are the mean ± SEM of eight different cultures performed in triplicate. **b** mRNA expression of cytokines (*light grey*), transcription factors (*dark grey*), and cytokine receptors (*black*) was determined by real-time PCR in activated/expanded CD4^+^CD45RO^+^ T cells on day 7 of culture, after PMA and ionomycin stimulation. Data were analyzed normalizing with β-actin mRNA expression and compared with the mRNA expression of CD4^+^CD45RO^+^ T cells from HD. The fold change of each mRNA expression with respect to different conditions is represented. Data are the mean ± SEM of eight different cultures performed in triplicate. Differences between CD4^+^CD45RO^+^ T cells cultured in the presence and the absence of VIP were statistically significant, *P < 0.05, **P < 0.01, ***P < 0.001

The presence of VIP during in vitro activation/expansion of memory Th cells reduces the gene expression of not only cytokines, but also transcription factors and cytokine receptors related to the Th17 subset and the pathogenic Th17/1 and Th1 phenotypes, with a greater intensity in HD than in eRA patients (Fig. 4). First, we performed correlation studies between the mRNA expression of master regulators of both Th17 and Th1 subsets. Results showed that, in memory Th cells from HD, RORC was positively correlated with Tbx21, while no correlation was observed in the presence of VIP (Fig. 4a). mRNA expression analysis showed that VIP significantly decreased IL-22, IL-2, STAT3, Tbx21, IL-12Rβ2 (main IL-12 receptor subunit involved in Th1 differentiation), IL-23R, and IL-21R in HD. However, VIP reduced IL-21, IL-2, and STAT3 expression in eRA patients (Fig. 4b). Next, we examined the effect of VIP on the secretion of several cytokines. The presence of VIP decreased IL-22 and GM-CSF levels in HD and IL-21 levels in eRA patients, whereas IL-9 was increased in HD (Table 1). Together, these data indicate that the presence of VIP during expansion/activation of memory Th cells induces a shift towards a non-pathogenic Th17 profile and decreases the Th1 and Th17/1 phenotypes, mainly in HD.

**Table 1:** Set of cytokines whose secretion is modified by VIP during activation/expansion of memory Th cells

	Healthy donors		eRA patients	
	−VIP	+VIP	−VIP	+VIP
IL-21	1.54 ± 0.20	1.17 ± 0.09	0.99 ± 0.08	0.53 ± 0.08***
IL-22	8.42 ± 1.22	4.84 ± 0.97*	6.54 ± 0.93	6.25 ± 1.16
GM-CSF	11.83 ± 1.37	6.46 ± 0.80*	9.73 ± 1.51	7.57 ± 1.38
IL-9	0.29 ± 0.06	0.85 ± 0.27*	0.19 ± 0.03	0.28 ± 0.08

Values are ng of cytokines/1.10^6^ cells. Data are the mean ± SEM of eight different cultures, performed in duplicate. Differences between CD4^+^CD45RO^+^ T cells cultured in the presence and the absence of VIP were statistically significant, *P < 0.05, ***P < 0.001

### Expression of VIP receptors in memory Th cells in eRA patients and HD

**Fig. 5:**
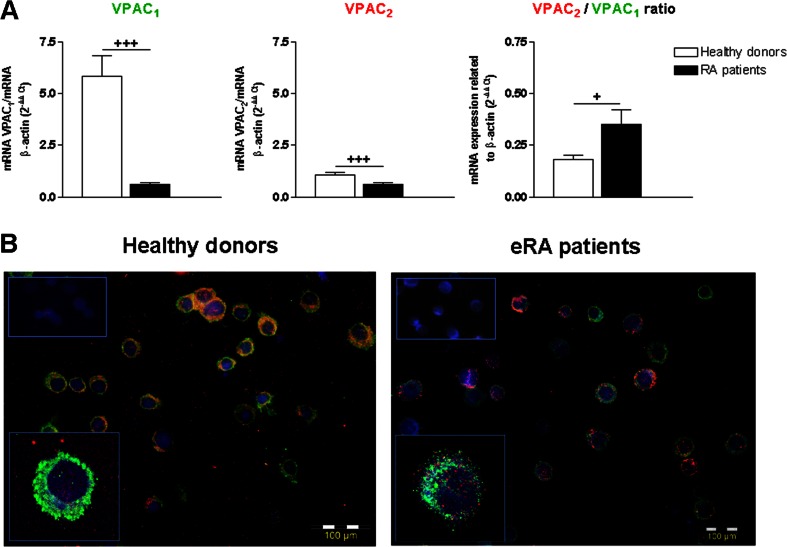
VIP receptor expression in memory Th cells from eRA patients and healthy donors. **a** The individual mRNA expression and the ratio between mRNA expression of VPAC_2_ and VPAC_1_ were determined by RT-PCR in activated/expanded memory Th cells on day 7 of culture after PMA and ionomycin stimulation. Data were analyzed normalizing with β-actin mRNA expression and compared with the VPAC_2_ mRNA expression of CD4^+^CD45RO^+^ T cells from healthy donors. Data are the mean ± SEM of eight different cultures performed in triplicate. Differences between CD4^+^CD45RO^+^ T cells from HD and eRA patients were statistically significant, ^+^ *P* < 0.05, ^+++^ *P* < 0.001. **b** Protein expression of VPAC_1_ and VPAC_2_ receptors were analyzed by immunofluorescence staining. Nuclei were counterstained with Hoechst (*blue*) and receptors were stained with Alexa Fluor 488 (*green*) and Alexa Fluor 594 (*red*) for VPAC_1_ and VPAC_2_, respectively. The figure shows a representative photo of activated/expanded memory Th cells from HD and eRA patients. Fluorescence was examined with an Olympus BX51 microscope with DP72 camera model (objective ×40). *Top left boxed areas* show a representative area of a negative control, performed in the absence of anti-VPAC_1_ or anti-VPAC_2_ antibodies. Fluorescence was examined with an Olympus BX51 microscope with DP72 camera model (objective ×40). *Bottom left boxed areas* show larger-magnification views of individual cells. Fluorescence was examined on a Leica SP-2 AOBS confocal microscope with inverted stand Leica DM IRE2 (objective ×63)

Although VPAC_1_ and VPAC_2_ receptors showed significantly lower mRNA expression, the VPAC_2_/VPAC_1_ ratio was higher in eRA patients (Fig. 5a). Additionally, we analyzed VPAC_1_ and VPAC_2_ protein expression in both HD and eRA patients by immunocytochemical analysis. As shown in Fig. 5b, both single stained (VPAC_1_ or VPAC_2_) and double-positive cells were found in HD and eRA patients.

## Discussion

To date, different studies have described the contribution of Th1 and Th17 cells to RA chronicity by controlling numerous cellular events [1, 3, 4, 25, 26]. However, these studies have been mostly performed in non-activated cells from fresh blood of RA patients. The present study analyzed the pathogenic Th17 and Th17/1 profile of memory Th cells after in vitro activation/expansion during 7 days in eRA patients and compared it to HD. Study in RA patients of recent onset allow to reproduce in vitro what might occur in patients with eRA in the active phase of the disease and avoid treatment interferences.

Th17 and Th1 involvement in RA is well established; however, it is less defined whether RA is mostly mediated by Th17 or Th1 cells or whether each subset is important in a different phase of the disease. Likewise, the presence of Th17 cells co-expresing IFNγ has been described in RA [13, 14]. Conversion of Th17 to Th1 phenotype might contribute to its pathogenic potential. Thus, in our studies we have characterized the presence of Th17, Th1, and Th17/1 cells within memory Th cells from eRA patients and compared it to HD after in vitro activation/expansion for 7 days. Percentage of RORC^+^ cells was higher than T-bet^+^ cells in both HD and eRA patients, indicating that Th17 cells were more abundant than Th1 cells. We further characterize the Th17 profile and higher frequencies of double-positive CCR6 and RORC cells were detected in eRA patients, in agreement with previous observations [9]. The CCR6 expression in Th17 cells suggests their ability to migrate to inflammatory sites [8] in response to an inflammatory microenvironment through the interaction with CCL20. Our proliferation studies on Th17 cells demonstrated that double-positive Ki67 and RORC cells were slightly increased in eRA patients. However, the percentage of double-positive Ki67 and T-bet cells were significantly decreased in eRA patients, suggesting that Th17 cells are more responsive than Th1 cells, in agreement with previous observations [9, 27]. This fact is further supported by the high expression of IL-17 in eRA patients. Moreover, mRNA expression of the transcription factors RORC, RORA, and STAT3 are higher in eRA patients. These results also suggest the functional increase of the Th17 cells in eRA patients, although their frequency in eRA patients is not significantly increased. In this regard, although it has been described an increased frequency of Th17 cells in peripheral blood of RA patients [4, 27], other studies have not confirm this increase [25]. Moreover, the hypothesis that the functional ability of Th17 cells is more important than their percentage in peripheral blood has been suggested [28]. Overall, we have found a stronger Th17 profile in activated/expanded memory Th cells from eRA patients than in HD, which agrees with the previously described important role of Th17 in RA [1, 3, 5]. IL-17 promotes the survival and proliferation of FLS in RA patients [6, 29] and in turn, FLS are able to control the pathogenicity of Th17 by modulating IL-17R and IL-23 expression [6, 29]. This feedback-loop mechanism could contribute to the synovium hyperplasia as well as to the inflammation in RA patients.

Recent reports indicate the existence of two types of functional Th17 cells depending on their cytokine secretion profile, pathogenic (IL-17, IL-21, IL-22, IL-2, IFNγ, and GM-CSF) and non-pathogenic cells (IL-17, IL-21, IL-9, and IL-10) [10, 11]. In addition, Th17 subset is also characterized by its phenotypic instability. Recent studies highlight the importance of their plasticity towards Th1 phenotype [12]. This phenomenon is even more relevant in RA patients given the implications of both subsets in the physiopathology of the disease [13, 14] and it has been suggested as one cause for the failure of therapies targeting Th17 cells [5]. Our results reported that the percentage of total T-bet-positive cells was lower in eRA patients. Given that the percentage of T-bet^+^/RORC^−^ cells was similar in both HD and eRA patients, this decrease in the T-bet percentage was due to the decrease in the percentage of T-bet^+^/RORC^+^ cells. It has been described that the frequency of IFNγ-producing CD4 T cells in peripheral blood from RA patients was lower compared to HD [28]. Likewise, it has also been shown no differences in the percentage of IFNγ^+^IL17^−^, IFNγ^+^IL17^+,^ and IFNγ^−^IL17^+^ in the peripheral blood of RA patients compared to HD [25]. Other authors have shown lower levels of Th17/1 cells in peripheral blood suggesting that it is due to the increase of their recruitment in the joint [13, 14]. On the other hand, results show a strong pathogenic and related to Th1 profile phenotype in eRA patients, showing higher levels of IFNу and GM-CSF cytokines and lower levels of IL-10 and IL-9 cytokines. High level of IL-2 and Tbx21 mRNA expression was also found in eRA patients. The discrepancy between the percentage of T-bet-expressing cells and the mRNA data related to T-bet expression could be due to a more T-bet transcription individually, although a lesser percentage of total T-bet-positive cells. On the other hand, this discrepancy could be also due to a post-transcriptional modulation of this molecule, for example by miRNAs or lcnRNAs. However, although lower T-bet-expressing cells were detected in eRA cells, these levels are sufficient to induce a greater IFNγ transcriptional response. In summary, our results indicate that activated/expanded memory Th cells from eRA patients show stronger pathogenic Th17 and Th17/1 profiles than cells from HD.

An endogenous mediator able to mediate the Th17 subset to a non-pathogenic phenotype and inhibit the Th17/1 and Th1 profiles would be a good candidate as a therapeutic agent in inflammatory diseases, specifically in RA. A recent study reported that patients who were unable to increase VIP levels in peripheral blood showed a worse clinical course despite receiving more intense treatment [30]. It is known that VIP modulates Th1, Th2, Th17, and Treg subsets [15, 16, 19, 23]. However, little is known about VIP modulation and VPAC receptor expression in Th lymphocytes from RA patients. We studied, for the first time, the effect of VIP on the Th17 heterogeneity and plastic profile of activated/expanded memory Th cells from eRA patients.

Regarding the Th17/1 and Th1 profiles, VIP reduced the Th1 profile in HD, which agrees with previous in vitro data in mouse and human cells [19, 21]. This neuroimmunopeptide was able to reduce the expression of two Th1 markers, Tbx21 and IL-12Rβ2, and to induce a lack of correlation between RORC and Tbx21 in HD. In addition, VIP down-regulated the gene expression of several Th17 and Th1 markers and pathogenic indicators including IL-22, IL-2, STAT3, IL-23R, and IL-21R in HD, but in eRA patients it only lowered IL-21, IL-2, and STAT3 expression. VIP also reduced IL-22 and GM-CSF production, as well as increased the IL-9 secretion, favoring the non-pathogenic profile in HD. IL-21 production was reduced in the presence of VIP in eRA patients. IL-21 has been implicated in RA [31] and blocking IL-21/IL-21R pathway showed an amelioration of the disease in RA animal models [32]. Moreover, IL-21 is a central memory T cell-associated cytokine that inhibits the generation of pathogenic Th1/17 effector cells [33]. NFkB is an important transcription factor for IL-21 [34] and VIP is able to reduce it in several cells [19]. Ex vivo studies have previously demonstrated the modulatory effect of VIP in RA [15–17]. In a pro-inflammatory milieu, VIP induced the down-regulation of IL-22-specific receptors in FLS, diminishing their potential to respond to pathogenic Th17 cells [35]. Therefore, our results confirm the VIP-mediated decrease in Th17 and Th1 cytokines reported in several inflammatory/autoimmune disease models [19, 21]. These VIP effects are mediated through its VPAC receptors. We demonstrated that VPAC_1_ and VPAC_2_ are expressed in activated/expanded memory Th cells. In eRA patients, the expression of both receptors is reduced in respect to HD, whereas the ratio of VPAC_2/_VPAC_1_ expression is higher. It has also been described that the presence and function of VPAC_2_ predominates over VPAC_1_ in FLS from RA patients [17]. This differential VIP receptor expression in eRA patients, and consequently different signal transduction, could also be involved in the weak effect of VIP their cells. Although, other explication of the smaller effect of VIP in eRA patients could be the differences in starting T cells, which are more pathogenic and associated to Th1 and Th17 phenotype.

Overall, our results indicate that activated/expanded memory Th cells from eRA patients generate a greater proportion of Th17 cells with a pathogenic Th17 and Th17/1 profile than those from HD. VIP lowered this pathogenic profile, being more important in HD. Findings presented here in RA patients with an early phase of the disease are very interesting from a therapeutic point of view and support that targeting molecules involved in the generation of pathogenic Th17, Th17/1, and Th1 phenotypes could have beneficial effects. Likewise, targeting VIP receptors could have a therapeutic effect through the reduction of these pathogenic subsets.
